# New insights in *Trichochloritis* Pilsbry, 1891 and its relatives (Gastropoda, Pulmonata, Camaenidae)

**DOI:** 10.3897/zookeys.865.36296

**Published:** 2019-07-22

**Authors:** Barna Páll-Gergely, Eike Neubert

**Affiliations:** 1 Plant Protection Institute, Centre for Agricultural Research, Hungarian Academy of Sciences, Budapest, Hungary Plant Protection Institute, Centre for Agricultural Research, Hungarian Academy of Sciences Budapest Hungary; 2 Natural History Museum of the Burgergemeinde Bern, Bernastr. 15, CH-3005 Berne, Switzerland Natural History Museum of the Burgergemeinde Bern Berne Switzerland; 3 Institute of Ecology and Evolution, University of Bern, 3012 Bern, Switzerland University of Bern Bern Switzerland

**Keywords:** Land snail, nomenclature, Southeast Asia, systematics, taxonomy

## Abstract

The genus *Bellatrachia* Schileyko, 2018 was described based on a specimen identified as Helix (Chloritis) pseudomiara Bavay & Dautzenberg, 1909. We concluded that the examined specimen is not that species, but *Helixcondoriana* Crosse & Fischer, 1863. Therefore, (1) the type species of *Bellatrachia* must be replaced with *Helixcondoriana*; (2) the species Helix (Chloritis) pseudomiara must be re-allocated to the genus *Trichochloritis*; (3) the erroneous treatment of the genus *Trichochloritis* by Schileyko (2007) needs to be corrected through the description of a new genus, *Dentichloritis***gen**. **nov.** based on *Helixbrevidens* Sowerby I, 1841. In addition, *Chloritismicrotricha* Möllendorff, 1898 is treated as a synonym of *Helixcondoriana*, and further information on the genitalia of *Chloritis* (?) *bifoveata* (Benson, 1856) is presented.

## Introduction

Almost 20 years ago, the second author of this work became fascinated by the enormously rich shell collection of Colonel Messager (see Breure and Páll-Gergely 2019) from northern Vietnam and Laos housed in the MNHN. While many type specimens taken from Messager’s collection were distributed through the activities of the describing authors to other institutions, the main body of the collection remained untouched in Paris. At the suggestion of the first author, we started to systematically compile data on the haired camaenid species of Southeast Asia.

This group was traditionally classified in the genera *Trichochloritis* Pilsbry, 1891 and *Trachia* E. von Martens, 1860 (Richardson 1985; Schileyko 2011; Wu et al. 2019); however, it was clear from the beginning that haired and non-haired shells are present in many camaenid genera, that the current classification is rather a paraphyletic “wastebasket taxon”, and that only the investigation of the morphology of the genital organs in combination with genetic data will recover the correct phylogenetic relationships. Nonetheless, even current modern research can add to the confusion rather than unravelling some of the old errors.

According to Schileyko (2007), the genus *Trichochloritis* consists of 10–12 species from southern China, Indochina Peninsula, and the Philippines. He published an illustration (drawing) of the shell of the type species, *H.breviseta* (Schileyko 2007: fig. 2032a), and added drawings of the reproductive anatomy of *H.brevidens* Sowerby I, 1841 (Schileyko 2007: fig. 2032b–c) as representative of *Trichochloritis*. However, the morphology of the genital organs of the latter species differs strongly from the conchologically similar genera as used here (*Trichochloritis*, *Bellatrachia*) from Continental Asia. In 2018, Schileyko described the monotypic genus *Bellatrachia*, a genus which was introduced based on conchological characters and traits of the genital anatomy of Helix (Chloritis) pseudomiara Bavay & Dautzenberg, 1909. Unfortunately, the anatomically examined specimen, which was collected in the Cat Tien National Park, southern Vietnam, was misidentified: in fact, Schileyko’s (2018) specimen is *Helixcondoriana* Crosse & Fischer, 1863.

These misidentifications and errors have nomenclatorial and taxonomical consequences: 1) the type species of *Bellatrachia* must be replaced; 2) the species Helix (Chloritis) pseudomiara Bavay & Dautzenberg, 1909 must be re-allocated in the genus *Trichochloritis*; 3) the erroneous treatment of the genus *Trichochloritis* by Schileyko (2007) needs to be corrected through the description of a new genus, *Dentichloritis* nov. gen. based on *Helixbrevidens* Sowerby I, 1841. In addition, the position of two continental species usually confined to *Chloritis* Beck, 1837, is discussed.

## Materials and methods

An ethanol-preserved specimen of *Chloritis* (?) *bifoveata* (Benson, 1856) was dissected under a Leica stereo microscope with a camera attachment to provide photographs of the external genital structure, from which drawings were produced. The inner structure of reproductive organs was illustrated from photographs.

Institutional abbreviations:

**BOR/MOL** BORNEENSIS collection of Institute for Tropical Biology and Conservation, Universiti Malaysia Sabah

**MNHN** Muséum National d’Histoire Naturelle (Paris, France)

**NHMUK** The Natural History Museum (London, UK)

**RBINS** Royal Belgian Institute of Natural Sciences (Brussels, Belgium

**SMF** Senckenberg Forschungsinstitut und Naturmuseum (Frankfurt am Main, Germany)

**ZMH** Zoological Museum, University of Hamburg (Germany)

**ZSI**Zoological Survey of India (Kolkata, India).

Abbreviations for anatomical details:

**EP** Epiphallus

**Fl** Flagellum

**MRP** Musculus retractor penis

**P** Penis

**Pa** Penial appendix

**RS** Receptaculum seminis

**VD** Vas deferens

## Taxonomy

### Family Camaenidae Pilsbry, 1893

#### Genus Bellatrachia

Taxon classificationAnimaliaStylommatophoraCamaenidae

Schileyko, 2018

4f0abe1a-5a83-402d-b0ca-bbae40f63549

Bellatrachia Schileyko, 2018: 169–171.

##### Type species.

Helix (Chloritis) pseudomiara Bavay & Dautzenberg, 1909 by monotypy.

The anatomically-examined specimen (i.e., on which the genus is based) was in fact *Helixcondoriana*. Under the provisions of Article 70.3 ICZN, we herewith replace the original type species Helix (Chloritis) pseudomiara Bavay & Dautzenberg, 1909 with *Helixcondoriana* Crosse & Fischer, 1863 as the type species of *Bellatrachia* Schileyko, 2018 to serve the stability of nomenclature.

##### Included species.

*Bellatrachiacondoriana* (Crosse & Fischer, 1863).

##### Diagnosis.

Shell depressed globular, apex not sunken, hairs or hair scars cover the entire shell. Penis rather long, subcylindrical, its inner surface bears longitudinal pilasters; penial verge absent; penial caecum absent; epiphallus slender, long, convoluted; retractor muscle attached at the penis-epiphallus transition; flagellum thick, with attenuated tip, approximately 2–2.5 times shorter than epiphallus; vagina slender, shorter than penis; stalk of bursa copulatrix long, with thickening at some distance from its origin, shape of bursa unknown (based on Schileyko 2018; see Fig. 4).

##### Description.

See *B.condoriana* below.

##### Remarks.

*Bellatrachia* differs from *Trichochloritis* in lacking the penial caecum.

#### Bellatrachia condoriana

Taxon classificationAnimaliaStylommatophoraCamaenidae

(Crosse & Fischer, 1863)

5074b943-e486-446e-89ee-f9d58a9bbdcc

Figs 1–2 , 3–4

Helix condoriana Crosse & Fischer, 1863: 351, pl. 14, fig. 1.
Chloritis microtricha Möllendorff, 1898: 71. syn. nov.
Chloritis (Trichochloritis) microtricha : Zilch 1966: 304, pl. 9, fig. 23.
Trichochloritis microtricha : Schileyko 2011: 47.
Trichochloritis condoriana : Schileyko 2011: 47.
Bellatrachia pseudomiara : Schileyko 2018: 169–171, figs 1–2 [non Helix (Chloritis) pseudomiara Bavay & Dautzenberg, 1909].

##### Type specimens.

*condoriana*: 1 syntype MNHN-IM-2000-1866, Poulo-Condor, D: 18.3 mm, H: 11.7 mm [this is the syntype figured in the original description]; *microtricha*: lectotype (selected by Zilch 1966) SMF 8540, Vietnam, Annam, Boloven, coll. O. Möllendorff ex coll. Roebelen, D: 24.8 mm, H: 17.7 mm.

##### Type locality.

“insula Poulo-Condor” [Con Son Island], Vietnam.

##### Additional specimens.

Vietnam, Bang-Kiang, coll. Denis ex Messager, MNHN-IM-2012-27151 (2 shells).

##### Diagnosis.

Shell biconvex with a whitish subsutural spiral, narrow umbilicus, and hair scars covering the entire surface.

##### Description.

Shell middle sized, biconvex, moderately thin-walled; last whorl only slightly expanding and descending abruptly towards aperture; colour dirty yellowish with a broad pale subsutural spiral band; whorls 4.5–5, separated by a rather shallow suture; body whorl faintly slightly angled; subsutural furrow shallow but present on the complete last whorl; protoconch consists of 1.25–1.5 whorls, very finely squamous, matte; the pattern of hair scars is dense and covers the complete teleoconch; aperture obliquely rounded, and the peristomal rims are close; peristome strongly expanded and somewhat reflected and reinforced by a white lip; parietal side with very thin, inconspicuous light layer; umbilicus open, of medium size, with blunt peripheral angulation, and partly covered by the columellar reflection.

##### Measurements.

D = 18.3–24.8 mm; H= 11.7–17.7 mm (n = 4).

##### Remarks.

**Figures 1–2.**
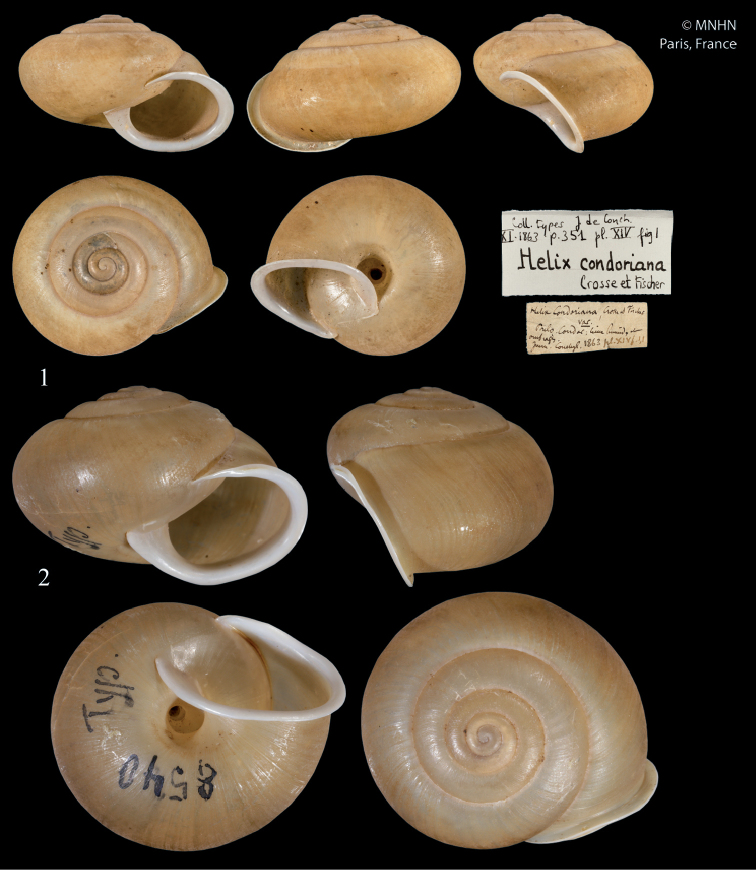
*Bellatrachiacondoriana***1** Syntype *Helixcondoriana* Crosse & Fischer, 1863, MNHN-IM-2000-1866, D = 18.3 mm, MNHN**2** lectotype *Chloritismicrotricha* Möllendorff, 1898, SMF 8540, D = 24.8 mm, S. Hof, Senckenberg. All photographs × 2.

**Figures 3–4.**
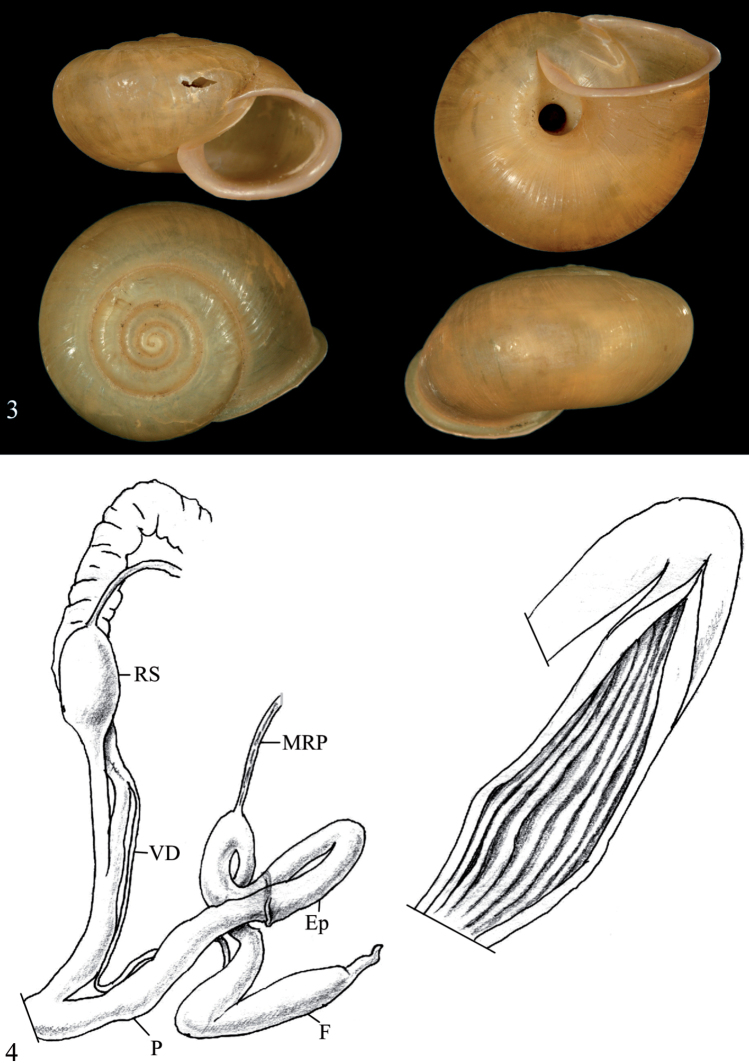
Original specimen “*Bellatrachiapseudomiara*” sensu Schileyko **3** shell (Schileyko 2018: 170, Fig. 1), D = 23.2 mm, A. Sysoev, photograph × 2 **4** Morphology of the genital organs of “*Bellatrachiapseudomiara*” sensu Schileyko (2018), modified after Schileyko (2018).

The syntype of *B.condoriana* (Fig. 1) is similar to the specimen identified as Helix (Chloritis) pseudomiara by Schileyko (2017) (Fig. 3), but the shell of the latter is somewhat more depressed. The shell of the lectotype of *B.microtricha* (Fig. 2) is larger and somewhat more globular that that of *B.condoriana*. However, both taxa agree quite well in other details such as the relative size of the umbilicus, formation of lip and aperture, and microsculpture of the teleoconch. In contrast, absolute dimensions proved to be insufficient traits for species-level distinction. Therefore, we consider *Chloritismicrotricha* as a synonym of *Bellatrachiacondoriana*. The subtle conchological differences in the shell morphology shown in Figs 1–3 may be part of the overall variation of *B.condoriana* or may signal a difference at the species level. This question can only be clarified by a revision of a larger number of specimens from the area.

#### Genus Trichochloritis

Taxon classificationAnimaliaStylommatophoraCamaenidae

Pilsbry, 1891

bb74dab8-810f-46d3-a5a4-89a76914850b

Trichochloritis Pilsbry, 1891: 267.
Trichochloritis : Schileyko 2007: 2113–2114, fig. 2032 (partim).

##### Type species.

*Helixbreviseta* L. Pfeiffer, 1862 by original designation.

##### Included species.

*Helixbreviseta* L. Pfeiffer, 1862, *Trachiapenangensis* Stoliczka, 1873.

##### Diagnosis.

Shell depressed globular, apex not sunken, hairs or hair scars cover the entire shell. Penis thickened, probably with penial verge (?) and a slender, relatively long penial caecum; epiphallus slender, shorter than penis; retractor muscle attached at the penis-epiphallus transition; flagellum short; vagina slender, shorter than penis; stalk of bursa copulatrix long, with thickened base and oval bursa (based on the drawings of Stoliczka 1873: plate 3, fig. 18 and Collinge 1903: plate 12, fig. 17.).

##### Remarks.

The anatomy of the genital organs of Helix (Trachia) malayana Möllendorff, 1887 (= *Trichochloritisbreviseta*; see Maassen 2001) was described by Collinge (1903), and that of *T.penangensis* is known from Stoliczka (1873), here re-drawn and provided in Fig. 8 (*penangensis*) and Fig. 9 (*breviseta*). Both species possess a penial caecum, which is here considered as a diagnostic trait for the genus. Without knowing the full anatomy, it is uncertain how many of the hairy *Chloritis*-like species of continental Asia belong to this group.

#### Trichochloritis breviseta

Taxon classificationAnimaliaStylommatophoraCamaenidae

(L. Pfeiffer, 1862)

46f356cb-b28e-4a32-97d2-7a42aaec783e

Figs 5–7 , 9 , 10

Helix breviseta L. Pfeiffer, 1862: 41–42, pl. 5, figs 4–5.
Helix (Trachia) malayana Möllendorff, 1887: 303.
Chloritis malayana Möllendorff, 1891: 335, pl. 30, figs 6–6a.
Helix (Trachia) malayana : Collinge 1903: 210, pl. 12, fig. 17.
Chloritis (Trichochloritis) malayana : Pilsbry 1893: 274, pl. 51, figs 34, 35.
Chloritis breviseta (and Chloritismalayana, which is considered a synonym): Maassen 2001: 120.
Trichochloritis breviseta : Schileyko 2011: 47.
Chloritis breviseta : Foon et al. 2017: 56, fig. 21C.

##### Type specimens examined.

*breviseta*: syntype MNHN-IM-2000-1847, Siam, D: 22.1 mm, H: 12.9 mm; *malayana*: syntypes (2 shells) NHMUK 1891.3.17.3–4, Perak, leg. Hungerford.

##### Additional specimens.

Perak, leg. Hungerford, NHMUK 1891.3.17.3–4 (2 shells of “*malayana*”); Larut, Malay Peninsula, NHMUK 1897.3.15.7 (1 shell of “*malayana*”); Malakka, Kelantan, Hochland v. Perak, coll. O. Möllendorff ex coll. H. Rolle ex coll. Waterstraat, SMF 8538/1 (“*malayana*”).

##### Type locality.

“Siam” (*breviseta*); “Perak” [Perak state, Malaysia] (*malayana*).

##### Diagnosis.

Shell depressed, unicoloured, yellowish, with permanent hairs; umbilicus funnel-shaped with a blunt peripheral angulation.

##### Description.

Spire only slightly elevated, shell depressed, shell thin; last whorl bluntly angled, a subsutural furrow is present but insignificant; colour yellowish, spiral band missing; the 4.5 whorls separated by a rather shallow suture; protoconch consists of slightly more than 1.5 whorls, squamous, bears minute wrinkled hair scars; teleoconch completely covered by a moderately dense pattern of hairs; bristles stiffy and durable and stick to the shell (their apical part breaks off, but a dark brown conical bristle cone is left making the surface of the shell quite rough); aperture subrectangular with only slightly oblique columella; peristome reflected and covered by a white lip; parietal region with very slight whitish, blunt lime layer, inconspicuous; columellar reflection small; umbilicus wide and funnel-shaped with a blunt peripheral keel.

**Figures 5–7.**
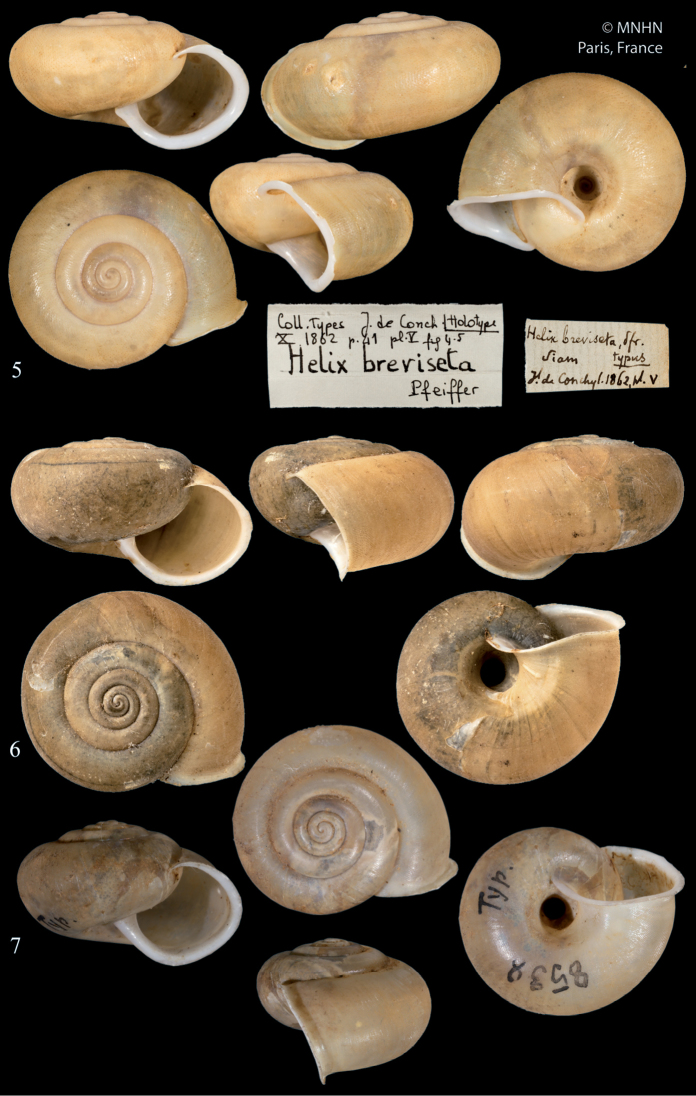
*Trichochloritisbreviseta***5** syntype *Helixbreviseta* L. Pfeiffer, 1862, MNHN-IM-2000-1847, D = 22.1 mm, MNHN**6** syntype Helix (Trachia) malayana Möllendorff, 1887, NHMUK 1891.3.17.3, D = 22.2 mm, NHMUK**7**SMF 8538 ex coll. Möllendorff, D = 20.6 mm, S. Hof, Senckenberg. All photographs × 2.

**Figures 8–9.**
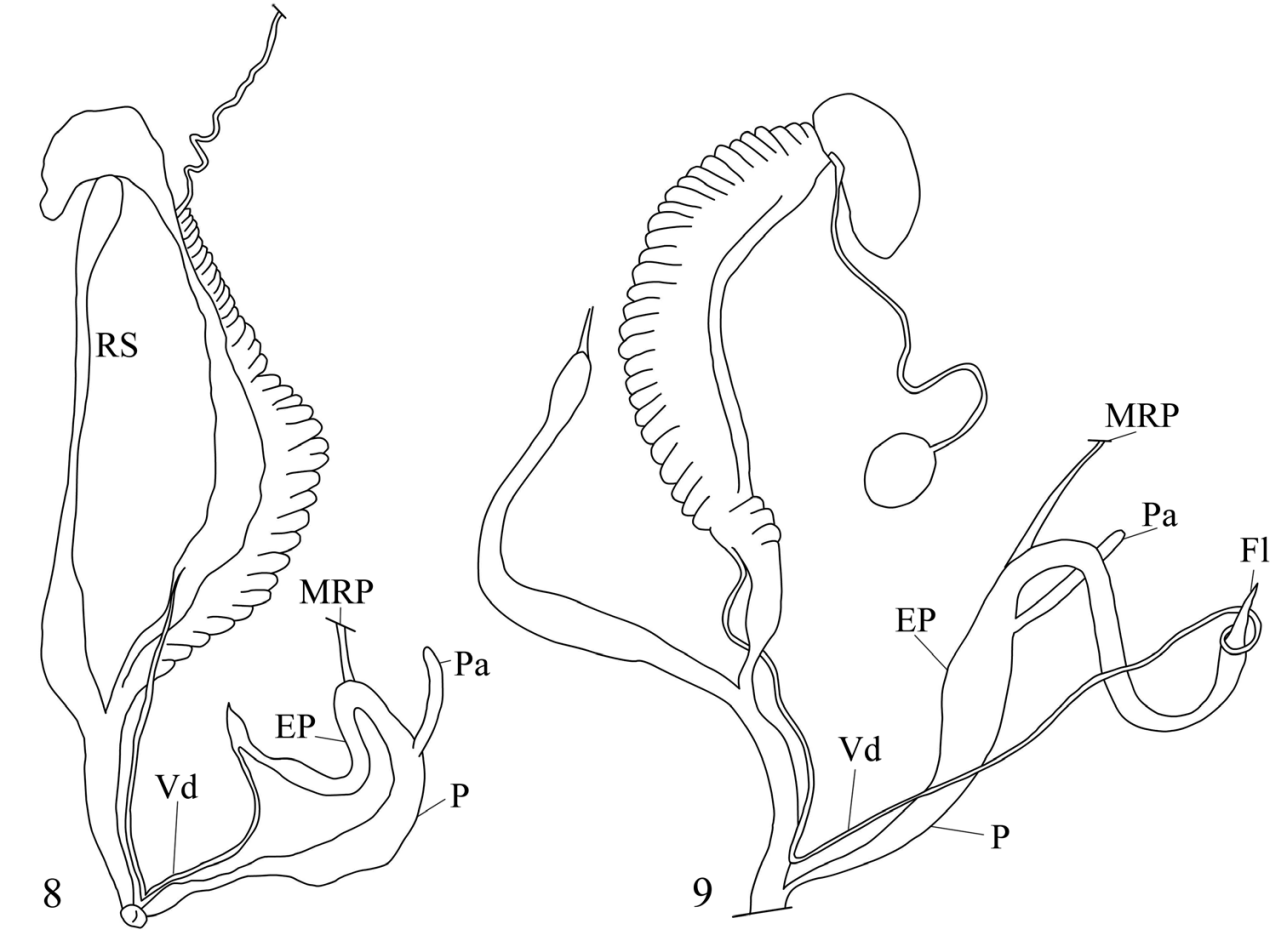
Morphology of the genital organs of *Trichochloritis* species **8***Trichochloritispenangensis* (Stoliczka, 1873) (redrawn from Stoliczka 1873) **9***Trichochloritisbreviseta* (L. Pfeiffer, 1862) (redrawn from Collinge 1903). Not to scale.

**Figures 10–12.**
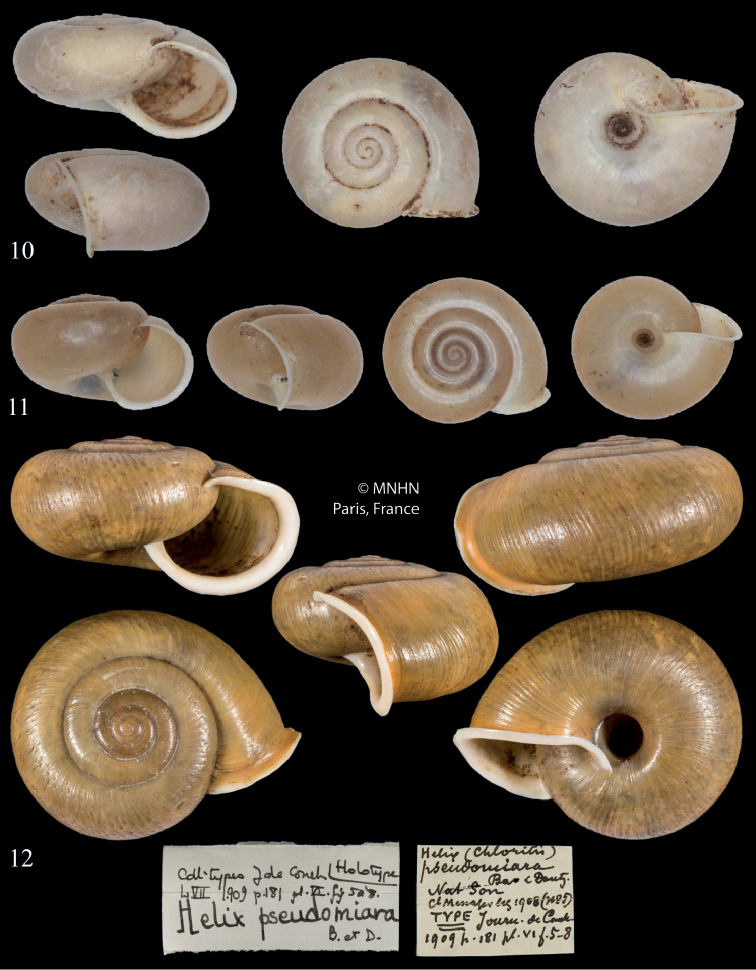
Shells of *Trichochloritis* species **10***Trichochloritisbreviseta*, BOR/MOL 9091, Perak, Ipoh, Gunung Kanthan plot, D = 19.5 mm **11***Trichochloritispenangensis*, BOR/MOL 11562, Perak, Ipoh, Gunung Pondok, plot, D = 16.2 mm **12***Trichochloritis* (?) *pseudomiara*, syntype of Helix (Chloritis) pseudomiara Bavay & Dautzenberg, 1909, D = 24.3 mm, MNHN. **10, 11** Junn Kitt Foon (published in Foon et al. 2017), all photographs × 2.

##### Measurements.

D = 22.9–24.1 mm; H = 12.9–14.7 mm (n = 4).

##### Distribution.

Malaysia and Thailand

#### Trichochloritis penangensis

Taxon classificationAnimaliaStylommatophoraCamaenidae

(Stoliczka, 1873)

7fea179d-a333-43ea-8f6a-158447e45fad

Figs 8 , 11

Trachia penangensis Stoliczka, 1873: 24–26, pl. 3, figs 1, 18–20.
Chloritis penangensis : Foon et al. 2017: 56–57, fig. 21D.

##### Type specimens.

The types should be in the Zoological Survey of India in Kolkata but were not found during a recent search (S.K. Sajan, pers. comm., December 2018). They were likewise not found in the NHM.

##### Type locality.

“Penang”.

##### Remarks.

“*Chloritispenangensis* has a much more globular shell with less expanded whorls compared to *Chloritisbreviseta* which has more expanded (perpendicular to the axis) whorls and thus, “wider” looking shells. These characters appear consistent for each species across Peninsular Malaysia (based on conchological comparisons), although shell size varies within each species.” (Junn Kitt Foon, pers. comm., 01 Dec 2018). To illustrate these differences, we illustrated the shells of both species (Figs 10, 11).

### Genus uncertain

#### Chloritis (?) bifoveata

Taxon classificationAnimaliaStylommatophoraCamaenidae

(Benson, 1856)

0c2e6b09-6c17-4534-9a86-2cb3b0552eef

Figs 15–17

Helix bifoveata Benson, 1856: 251.
Chloritis bifoveata : Sutcharit and Panha 2010: 278–283, figs 1A, B, 2A–F, 3A–D, table 1.

##### Specimens examined.

Thailand: Krabi: Phanom Benja National Park, Huai To waterfall and surrounding rain forest, 120 m, 08°14'21"N, 098°54'52"E, 08°14'08"N, 098°55'12"E, leg. Hausdorf, 28.07.2010, ZMH 51997/2.

##### Remarks.

For a detailed description of the shell refer to Sutcharit & Panha, 2010. Our data on the reproductive anatomy largely matches that of Sutcharit and Panha (2010), with the following two exceptions: the flagellum is relatively long and slender, and the penial verge is not irregularly shaped but conical and deeply grooved with the folds starting from the epiphallus.

#### Trichochloritis (?) pseudomiara

Taxon classificationAnimaliaStylommatophoraCamaenidae

 (Bavay & Dautzenberg, 1909)

80fabb3f-e17c-43f4-a52b-59f18f17c4f6

Fig. 12

Helix (Chloritis) pseudomiara Bavay & Dautzenberg, 1909a: 236; Bavay & Dautzenberg 1909b: 181, pl. VI, figs 5–8.
Trachia pseudomiara : Schileyko 2011: 45.

##### Type specimens examined.

syntype MNHN-IM-2000-31774, Nat Son, leg. Messager, D: 24.3 mm, H: 13.3 mm.

##### Other specimens examined.

Muong-Hum, RBINS/1; Muong-Hum, leg. Messager 1908, RBINS/1; Tonkin, Phong-Tho, RBINS/1 (mixed sample with *Trichochloritis* sp.); Nat-Son, RBINS/3 (mixed sample with *Trichochloritis* sp.); Tonkin, leg. Messager, RBINS/22 (some of them are juveniles); Tonkin, Phong-Tho, NHMUK 1909.7.9.57/1 (photographed for the North Vietnamese Land Snail Guide); Tonkin, Muong-Hum, MNHN-IM-2012-27105/2; Vietnam, Cam Duong, MNHN-IM-2012-27106/2 (probably erroneous locality?); Tonkin, Phong-Tho, MNHN-IM-2012-27107/2; Tonkin, Muong-Hum, MNHN-IM-2012-27108/2; Tonkin, Muong-Hum, leg. Messager, MNHN-IM-2012-27109/2; Tonkin, MNHN-IM-2012-27110/1; Haut Tonkin, MNHN-IM-2012-27111/1.

##### Type locality.

Vietnam, N Vietnam: Nat Son.

##### Diagnosis.

A rather large, usually dark species with rounded body whorl, fine radial growth lines and deep hair scars; umbilicus open, only a small part of it is covered by the columellar reflection.

##### Description.

Shell rather large, almost flat, with relatively thick wall; body whorl rounded; last half whorl with or without very shallow subsutural furrow; the 4.75–5.25 whorls are separated by a shallow suture; colour greyish yellowish, or brown to olive green; protoconch consists of 1.5 whorls, finely granulate, with fine radial lines near the suture of the last half whorl; teleoconch finely, irregularly wrinkled, and covered with very deep hair scars, which are visible to the naked eye as well on the body whorl; hairs not permanent, although we did not have access to live collected specimens; aperture ovoid; peristome expanded and slightly reflected, and reinforced by a thickened whitish/light brown lip; parietal region with an inconspicuous layer, which is often darker than the rest of the shell; umbilicus widely open, concave and funnel-shaped, slightly covered by reflected peristome.

##### Measurements.

D = 21.3 –26.0 mm, H = 11.8–14.4 mm (n = 3).

##### Distribution.

This species is known only from the northernmost part of Vietnam, along the Chinese border.

##### Remarks.

This species can easily be identified based on the dark green-coloured shell and the deep, widely spaced hair scars that cover the entire teleoconch.

#### Genus Dentichloritis gen. nov.

Taxon classificationAnimaliaStylommatophoraCamaenidae

c031568d-280e-480f-ba4b-07842955d14c

http://zoobank.org/16A52E49-1C90-47D6-B66A-56F12C02B11A

Trichochloritis : Schileyko, 2007: 2113–2114, fig. 2032a–c (partim).

##### Type species.

*Helixbrevidens* Sowerby I, 1841: 25 (Puerto Galero, Philippines).

##### Diagnosis.

Shell depressed globular, apex not sunken, hairs or hair scars cover the entire shell, aperture with a basal denticle. Penis very thick-walled, with narrow lumen, internally with very large conic tubercles in main chamber; flagellum and epiphallus absent; vas deferens passes gradually enlarging into penis; retractor muscle inserts at curvature of vas deferens close to its joint with penis; penial sheath thin, surrounds upper two third part of penis; vagina shorter than penis, thick.

##### Etymology.

The name *Dentichloritis* refers to the presence of a denticle on the basal peristomal lip and the conchological similarity to *Chloritis*.

##### Remarks.

There are seven *Trichochloritis* species known from the Philippines (Richardson 1985), and four of them have been photographed by Zilch (1966). They differ from *D.brevidens* in the open umbilicus and the lack of denticle on the basal lip. Therefore, we retain them in *Trichochloritis* until ethanol-preserved specimens become available.

#### Dentichloritis brevidens

Taxon classificationAnimaliaStylommatophoraCamaenidae

(Sowerby I, 1841)

0a370ad6-5c23-45be-9633-e139a36e28f1

Figs 13–14

Helix brevidens Sowerby I, 1841: 25.
Trichochloritis brevidens : Schileyko 2007: 2113–2114, fig. 2032b, c.

##### Type specimens examined.

**Figures 13–14.**
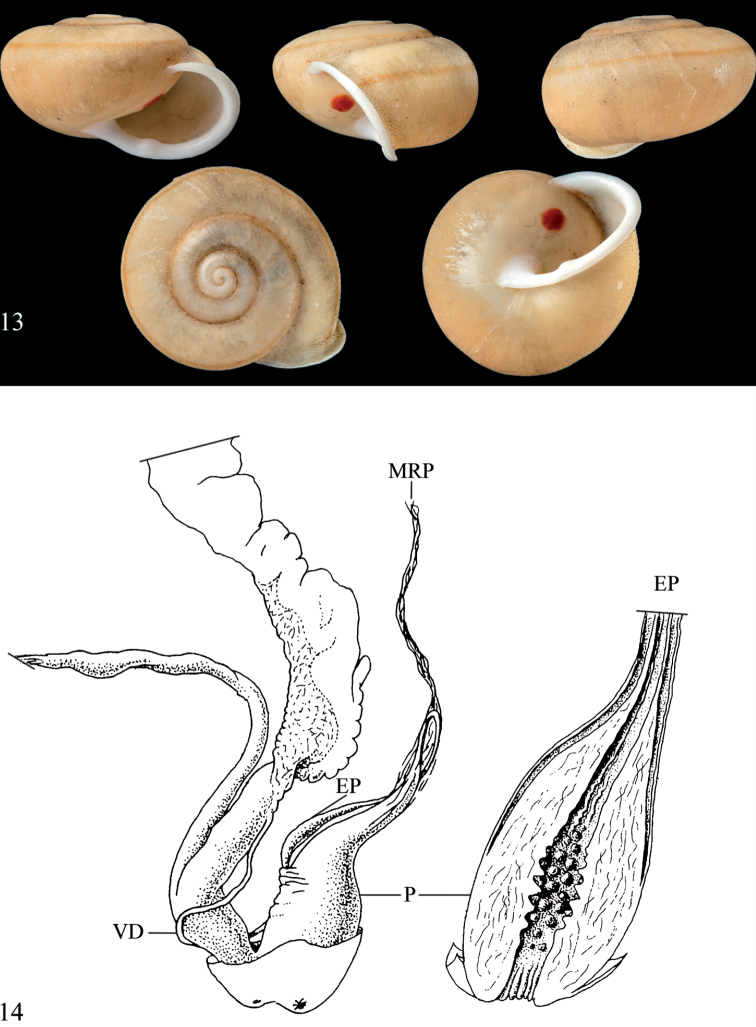
*Dentichloritisbrevidens***13** syntype *Helixbrevidens* Sowerby I, 1841, NHMUK 20190452, D = 19.5 mm **14** Morphology of the genital organs of *Dentichloritisbrevidens*; modified after Schileyko (2007).

Philippines, m.c. (Museum Cuming), 3 syntypes NHMUK 20190452 (D of photographed shell = 19.5 mm [Fig. 13]).

##### Type locality.

Philippines, Puerto Galero (Municipality of Puerto Galera, municipality in the province of Oriental Mindoro).

##### Diagnosis.

A middle-sized, yellowish species with a slender reddish peripheral belt, short hairs on the entire shell, nearly closed umbilicus (only visible in oblique view), and a slight thickening (denticle) on the basal part of peristome.

##### Description.

Shell medium sized, depressed globular; body whorl rounded with slight indication of a blunt shoulder; last quarter to half whorl with a very shallow subsutural furrow; the 3.75–4 whorls are separated by a shallow suture; colour yellowish to ochre with a reddish slender belt above shoulder (midpoint of body whorl); protoconch consists of 1.5–1.75 whorls, finely granulate, with fine radial wrinkles; teleoconch covered by short hairs or hair scars, which are visible to the naked eye as well; aperture semilunar; peristome expanded and slightly reflected, and reinforced by a thickened whitish brown lip; a slight swelling (denticle) visible on basal part of peristome, between the midpoint of the basal peristome and the columella; parietal region with an inconspicuous layer, which is matter than the rest of the shell; umbilicus nearly closed by columellar reflection, visible only by oblique view.

Anatomy: Penis very thick-walled, with narrow lumen, internally with short plicae in basal part and very large conic tubercles in main chamber; flagellum and epiphallus absent; vas deferens rather long, evenly thin down to atrium; approximately one third way up it is attached to penis, and after penis is enlarged and fusiform, then in becomes very thin, thread-like, forming a sharp curvature and passes to penis, gradually enlarging; penial retractor attached to curvature of vas deferens and continues as a fine membrane down to middle part of penis; penial sheath thin, surrounds upper two third part of penis. Vagina shorter than penis, thick; spermatheca without visible division to stalk and reservoir, not attending albumen gland and provided with apical ligament (based on Schileyko 2007: 2113–2114, fig. 2032b, c).

**Figures 15–17.**
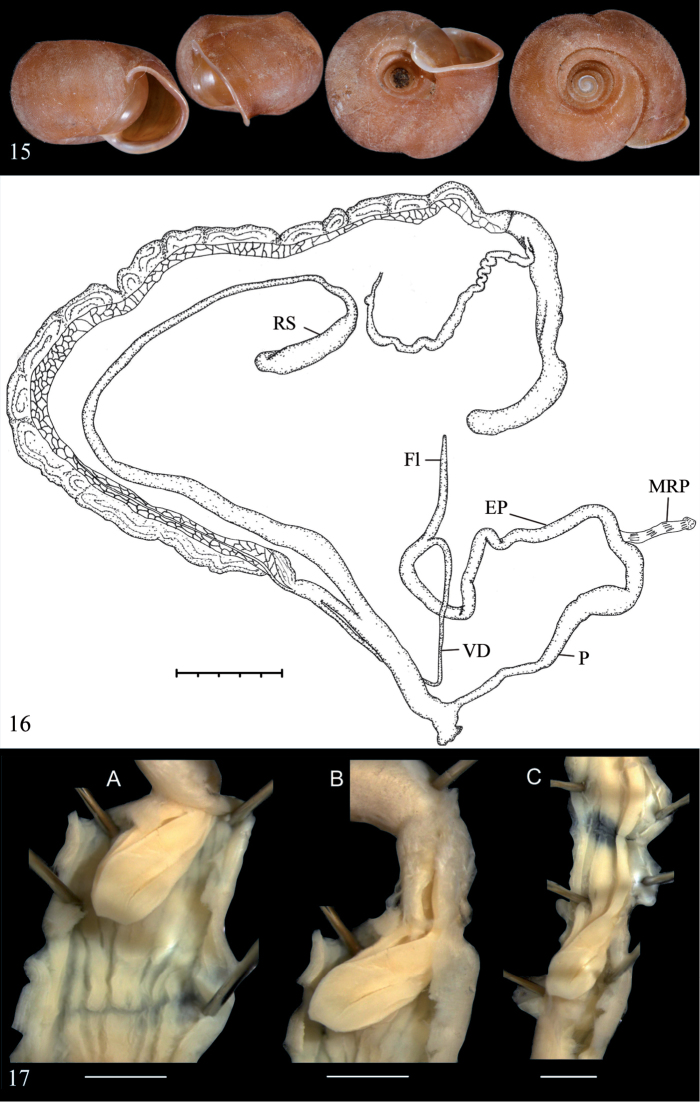
*Chloritis(?)bifoveata***15** shell of dissected specimens of *Chloritisbifoveata* (Thailand: Krabi: Phanom Benja National Park, ZMH 51997) **16** Situs of its genital organs **17** Penial verge of *Chloritisbifoveata*. A: verge visible from penis lumen; B: starting to penial verge from epiphallus; C: epiphallus opened until penial verge. Scale bar 1 mm.

## Discussion

Based on an anatomically examined specimen from southern Vietnam identified as *Helixpseudomiara* Bavay & Dautzenberg, 1909, Schileyko (2018) described the genus *Bellatrachia* Schileyko, 2018. However, that specimen is clearly incorrectly identified. Schileyko’s (2018) specimen has a rounded aperture and fine hair scars with fine silky periostracum. Thus, it closely resembles *Helixcondoriana* Crosse & Fischer, 1863, also known from southern Vietnam. In contrast, the true *Helixpseudomiara* is known only from northern Vietnam, and its shell has characteristic deep and sparsely arranged hair scars. Furthermore, the aperture of the latter is rather oval, not rounded. The reproductive anatomy of type species of *Trichochloritis* Pilsbry, 1891, *Trichochloritisbreviseta* (L. Pfeiffer, 1862), was described by Collinge (1903). Although it is not sufficiently detailed (i.e., the inner structure of penis is unknown), it is useful enough to diagnose *Trichochloritis*. The anatomy of *Trichochloritispenangensis* (Stoliczka, 1873) was described in the original generic description, and it largely matches with that of *T.breviseta*. Schileyko (2007) described the genitalia of *Trichochloritisbrevidens* (Sowerby I, 1841), originally described from Mindoro Island, the Philippines, as a representative of *Trichochloritis*. The reproductive anatomy of that species, however, differs from those of continental (true) *Trichochloritis* in several important characters. Therefore a new genus, *Dentichloritis* gen. nov. is erected for *T.brevidens*. The largely different anatomy, together with biogeographical reasons, suggest that *Trichochloritis* (continental Asia) and *Dentichloritis* gen. nov. (Philippines) are probably not even closely related.

In the original description of *Trichochloritis*, Pilsbry (1891) claimed that the most closely related genus was *Planispira* Beck, 1837. The anatomy of the type species of that genus (*Helixzonaria* Linnaeus, 1758) was described by Schileyko (2003), and is distinguished from *Trichochloritis* at first sight by the absence of a penial caecum.

It is difficult to interpret the relationship of *Trichochloritis* with *Chloritis*, because the reproductive anatomy of the type species of the latter (*Helixungulina* Linnaeus, 1758, by subsequent designation of Martens in Albers, 1860, from Ceram Island, Indonesia) is unknown. *Chloritis* is diagnosed conchologically mainly based on the sunken spire and the hairless shell (Schileyko 2003). Thus, the two continental species assigned to *Chloritis*, namely *Chloritisbifoveata* (Benson, 1856) and *Chloritisdiplochone* Möllendorff, 1898, do not even fit due to their strongly hairy shells. It is very unlikely that the two species inhabiting Thailand and Malaysia would belong to the same group as a species from Ceram Island. However, we refrain from erecting a genus for *C.bifoveata* and *C.diplochone* until we have more information on the anatomy of *C.ungulina*.

## Supplementary Material

XML Treatment for Bellatrachia

XML Treatment for Bellatrachia condoriana

XML Treatment for Trichochloritis

XML Treatment for Trichochloritis breviseta

XML Treatment for Trichochloritis penangensis

XML Treatment for Chloritis (?) bifoveata

XML Treatment for Trichochloritis (?) pseudomiara

XML Treatment for Dentichloritis

XML Treatment for Dentichloritis brevidens

## References

[B1] Albers JC (1860) Die Heliceen nach natürlicher Verwandtschaft systematisch geordnet. Zweite Ausgabe, nach dem hinterlassenen Manuskript besorgt von Eduard von Martens. Wilhelm Engelmann, Leipzig, 359 pp 10.5962/bhl.title.11218

[B2] Bavay A Dautzenberg P (1909a) Molluscorum terrestrium tonkinorum diagnoses. Journal de Conchyliologie 56: 229–251. [“1908”]. https://biodiversitylibrary.org/page/16298225

[B3] Bavay A Dautzenberg P (1909b) Description de Coquilles Nouvelles de l’Indo-Chine. Journal de Conchyliologie 57: 81–105, 163–206, 279–288. https://biodiversitylibrary.org/page/27393324

[B4] Beck H (1837) . Index molluscorum praesentis aevi musei principis augustissimi Christiani Frederici. Hafniae, Copenhagen, 99 pp 10.5962/bhl.title.77331

[B5] Benson WH (1856) Descriptions of one Indian and nine new Burmese Helices; and notes on two Burmese Cyclostomacea Annals and Magazine of Natural History, Series 2 18: 249–254. 10.1080/00222935608697626

[B6] Breure ASH Páll-Gergely B (2019) More than just a name: Colonel Messager and his correspondents. Zoosystema 41(2): 7–19. 10.5252/zoosystema2019v41a2

[B7] Collinge WE (1903) : Report on the non-operculate land Mollusca. Fasciculi Malayenses, Zoology 2: 205–218. https://biodiversitylibrary.org/page/52164094

[B8] Crosse H Fischer P (1863) Note sur la faune malacologique de Cochinchine, comprenant la description des espèces nouvelles ou peu connues. Journal de Conchyliologie 11: 343–379. https://biodiversitylibrary.org/page/15338393

[B9] Foon JK Clements GR Liew T-S (2017) Diversity and biogeography of land snails (Mollusca, Gastropoda) in the limestone hills of Perak, Peninsular Malaysia. ZooKeys 682: 1–94. 10.3897/zookeys.682.12999 PMC5523159 28769723

[B10] Maassen WJM (2001) A preliminary checklist of the non-marine molluscs of West Malaysia. De Kreukel (extra edition 2001) 2001: 1–155.

[B11] Möllendorff OF von (1887) The landshells of Perak. The journal of the Asiatic Society of Bengal 55(2): 299–316. https://biodiversitylibrary.org/page/35545670

[B12] Möllendorff OF von (1891) On the land and freshwater shells of Perak. Proceedings of the Zoological Society of London 1891: 330–348. 10.1111/j.1096-3642.1891.tb01757.x

[B13] Möllendorff O (1898) Die Binnenmollusken Annams. Nachrichtsblatt der Deutschen Malakozoologischen Gesellschaft 30(5 & 6): 65–85. https://biodiversitylibrary.org/page/28228483

[B14] Pfeiffer L (1862) Diagnoses de neuf espèces nouvelles provenant de Siam. Journal de Conchyliologie 10: 39–6. https://biodiversitylibrary.org/page/15134735

[B15] Pilsbry HA (1890–1891) Manual of Conchology, (2) 6. Helicidae, vol. IV. Academy of Natural Sciences, Conchological Section, Philadelphia, 324 pp https://biodiversitylibrary.org/page/23626788

[B16] Pilsbry HA (1892–1893) Manual of Conchology, (2) 8. Helicidae, vol. VI. Academy of Natural Sciences, Conchological Section, Philadelphia, 314 pp https://biodiversitylibrary.org/page/1287448

[B17] Pilsbry HA (1893–1895) Manual of Conchology, (2) 9. Helicidae, vol. VII. Guide to the study of Helices. Academy of Natural Sciences, Conchological Section, Philadelphia, 366 + 126 pp. https://biodiversitylibrary.org/page/1102607

[B18] Richardson L (1985) Camaenidae: Catalog of species. Tryonia, Miscellaneous Publications of the Department of Malacology of the Academy of Natural Sciences of Philadelphia 12: 1–479.

[B19] Schileyko AA (2003) Treatise on recent terrestrial pulmonate molluscs. Part 11. Trigonochlamydidae, Papillodermidae, Vitrinidae, Limacidae, Bielziidae, Agriolimacidae, Boettgerillidae, Camaenidae. Ruthenica, Supplement 2(11): 1467–1626.

[B20] Schileyko AA (2007) Treatise on Recent terrestrial pulmonate molluscs, Part 15 Oopeltidae, Anadenidae, Arionidae, Philomycidae, Succineidae, Athoracophoridae. Ruthenica, Supplement 2(15): 2049–2209.

[B21] Schileyko AA (2011) Check-list of land pulmonate molluscs of Vietnam (Gastropoda: Stylommatophora). Ruthenica 21(1): 1–68.

[B22] Schileyko A (2018) On the genus *Trachia* auct. (Gastropoda, Pulmonata, Camaenidae). Ruthenica 28(4): 169–174.

[B23] Sowerby GB I (1841) Descriptions of new species of the family Helicidae, collected by Mr. H. Cuming in the Philippine Islands (continuation). Proceedings of the Zoological Society of London 1841: 24–26. https://biodiversitylibrary.org/page/30679646

[B24] Stoliczka F (1873) On the land-shells of Penang Island, with descriptions of the animals and anatomical notes; part second, Helicacea. Journal of the Asiatic Society of Bengal 42: 11–38. https://biodiversitylibrary.org/page/35545891

[B25] Sutcharit C Panha S (2010) Taxonomic re-evaluation of *Chloritisbifoveata* (Benson 1856) and *C.diplochone* Möllendorff 1898 (Pulmonata: Camaenidae). Journal of Conchology 40(3): 277–285.

[B26] Wu M Chen Z Zhu X (2019) Two new camaenid land snails from Central China (Eupulmonata, Camaenidae). ZooKeys 861: 129–144. 10.3897/zookeys.861.35430 31335921 PMC6629712

[B27] Zilch A (1966) Die Typen und Typoide des Natur-Museums Senckenberg, 35: Mollusca, Camaenidae (5). Archiv für Molluskenkunde 95(5/6): 293–319, Taf. 7–11.

